# Non-Hodgkin’s lymphoma classification using 3D radiomics machine learning models for precision imaging in oncology

**DOI:** 10.1186/s12880-025-02006-3

**Published:** 2025-10-30

**Authors:** Christoph G. Lisson, Michael Götz, Daniel Wolf, Sabitha Manoj, Luisa Gallee, Stefan A. Schmidt, Eugen Tausch, Christof Schneider, Stephan Stilgenbauer, Ambros J. Beer, Meinrad Beer, Nico Sollmann, Catharina S. Lisson

**Affiliations:** 1 Department of Diagnostic and Interventional Radiology, University Hospital Ulm, Albert-Einstein-Allee 23, 89081 Ulm, Germany; 2 Center for Translational Imaging “From Molecule to Man” (MoMan), Department of Internal Medicine II, University Hospital Ulm, Ulm, Germany; 3 Center for Personalized Medicine (ZPM), University Hospital Ulm, Ulm, Germany; 4 Artificial Intelligence in Experimental Radiology (XAIRAD), Department of Diagnostic and Interventional Radiology, University Hospital Ulm, Ulm, Germany; 5 Department of Internal Medicine III, University Hospital Ulm, Ulm, Germany; 6 Comprehensive Cancer Center Ulm (CCCU), University Hospital Ulm, Ulm, Germany; 7 Department of Nuclear Medicine, University Hospital Ulm, Ulm, Germany; 8 Division Medical Image Computing, German Cancer Research Center (DKFZ), Heidelberg, Germany

**Keywords:** Non-Hodgkin lymphoma, Lymph nodes, Radiomics, Machine learning, Precision oncology, Imaging biomarkers.

## Abstract

**Purpose:**

To apply quantitative imaging analysis for noninvasive classification of the most frequent subtypes of Non-Hodgkin Lymphoma (NHL) as a basis for a clinical imaging genomic model to support therapeutic monitoring and clinical decision making.

**Materials and methods:**

In this single-center study, 201 treatment-naïve patients with biopsy-proven NHL (50 diffuse large B-cell lymphoma [DLBCL], 51 mantle cell lymphoma [MCL], 49 follicular lymphoma [FL], and 51 chronic lymphocytic leukemia [CLL]) and 39 treatment-naïve non-small cell lung cancer patients with positron emission tomography (PET)/computed tomography (CT)-confirmed healthy axillary lymph nodes (LNs) were retrospectively analyzed. Three-dimensional (3D) segmentation and radiomic analysis of pathologically enlarged nodes (*n* = 1,628) were performed on contrast-enhanced CT scans, including healthy LNs as references. Feature selection was performed using a random forest (RF) classifier. Multiclass Classifier was performed using a Light Gradient Boosting Machine (LGBM) classifier for lymphoma subtype classification.

**Results:**

Performance to classify lymphoma from non-lymphoma and lymphoma subtypes was as follows: lymphoma vs. non-lymphoma: area under the curve (AUC) = 0.999; MCL vs. other NHL: AUC = 0.997; DLBCL vs. other NHL: AUC = 0.971; CLL vs. other NHL: AUC = 0.956; FL vs. other NHL: AUC = 0.892.

**Conclusion:**

Radiomics combined with multiclass machine learning enables highly accurate, non-invasive differentiation of the major NHL subtypes on routine contrast-enhanced CT. By reliably separating indolent from aggressive phenotypes, this approach lays the groundwork for imaging-genomic models that could streamline biopsy guidance, enhance therapeutic monitoring, and advance precision oncology in lymphoma care.Conducted as a single-centre, retrospective proof-of-concept with internal patient-level cross-validation, these results are promising and form the basis for a prospective multicentre study to confirm generalisability and clinical utility.

**Clinical relevance statement:**

Accurate lymphoma classification is essential for predicting clinical behavior and guiding treatment. Imaging aids in disease staging, with quantitative analysis showing promise in predicting pathology and outcome. We explored machine learning on imaging features for lymphoma classification, thus enhancing clinical decisions.

**Supplementary Information:**

The online version contains supplementary material available at 10.1186/s12880-025-02006-3.

## Introduction

Non-Hodgkin lymphoma (NHL) is a heterogeneous group of hematologic neoplasms, with an incidence of 553,000 new cases and 250,000 deaths attributed to NHL in 2022,

 [1]. The 5th edition of the World Health Organization (WHO) classification of tumors of hematopoietic and lymphoid tissues delineates over 100 distinct categories of B- and T-cell lymphomas with diffuse large B-cell lymphoma (DLBCL) being the most common aggressive type of NHL and follicular lymphoma (FL) the most common indolent NHL in Western countries [2, 3]. Accurate classification of lymphoma subtypes is essential for predicting clinical outcomes and guiding treatment decisions [4, 5]. Because of the large number of different subtypes of lymphoma, inter-tumoral heterogeneity between subtypes is very common and the same subtype of lymphoma can exhibit different intra-tumoral heterogeneity within the same patient [6, 7].

The classic example of intra-tumoral heterogeneity is the transformation of a low-grade lymphoma into a high-grade lymphoma, which is associated with disease progression and poor prognosis [8, 9]. Thus, it is important to explore the heterogeneity of lymphoma, and to specifically define a “clinical-imaging-genomic model”.

Although histopathological examination of resected lymph nodes remains the reference standard for definitive subtyping, it is invasive, costly, and often impractical in routine care [10]. Consequently, non-invasive imaging modalities — CT, MRI, and 18 F-fluorodeoxyglucose positron emission tomography (FDG-PET) — have become indispensable for initial staging and treatment planning in NHL [11].

Driven by the demands of precision medicine, these techniques have progressed beyond mere visual assessment to constitute a rich repository of quantitative data amenable to advanced analytical methods (Gillies, Kinahan & Hricak 2016) [12].

Radiomics is a method for extracting quantitative imaging characteristics from diagnostic medical images, known as radiomic features [13].

These radiomic features, including textural features that capture spatial signal intensity or gray-scale patterns, allow assessing tumor heterogeneity [14, 15].

This heterogeneity is closely related to tumor biology and can have an impact on disease progression, therapeutic response, and clinical outcome [16, 17]. Radiomics has shown promising results to analyze tumour heterogeneity using different imaging techniques, including artificial intelligence-based machine-learning algorithms [18–21].

Although advanced imaging techniques can already distinguish lymphoma from other malignancies, subtype-level classification remains an unmet need: the few radiomics studies published to date typically address only one or two NHL entities [22–30].

In addition, there are no studies that have used CT texture analysis of enlarged LNs to classify more than two lymphoma subtypes or that have included healthy LNs as a reference.

Therefore, we aimed to investigate whether it is possible to use radiomics-based machine learning prediction models on CT images for the classification of healthy LNs from lymphoma, and to classify the most common NHL subtypes, namely diffuse large B-cell lymphoma (DLBCL), follicular lymphoma (FL), chronic lymphocytic leukemia (CLL), and mantle cell lymphoma (MCL).

Accordingly, this study was deliberately designed as a proof-of-concept feasibility analysis with patient-level cross-validation to demonstrate signal detectability; the encouraging results provide a strong rationale for prospective, multicentre validation under real-world prevalence.

## Materials and methods

### Study design and patients

A total of 201 treatment-naïve patients with histopathologically proven NHL (according to biopsy) were included in this monocentric retrospective analysis (Table 1): 51 patients with CLL (35 male; mean age 59.0 ± 10.9 years), 51 with MCL (43 male; mean age 60.5 ± 9.5 years), 50 with DLBCL (25 male; mean age 64.5 ± 12.2 years), and 49 with FL (25 male; mean age 61.2 ± 13.3 years).

**Table 1:** Baseline demographics of the malignant lymphoma cohort and the non-lymphoma cohort

Characteristics	MCL	DLBCL	FL	CLL	Non- Lymphoma
Cases (Percentage of Lymphoma Cohort)	51 (25,4%)	50 (24,9%)	49 (24,4%)	51 (25,4%)	39
Age (in Years) Mean ± Standard Deviation	59.0 ± 10.9	64.5 ± 12.2	61.2 ± 13.3	59.0 ± 10.9	64.9 ± 8.5
Male	43 (84.3%)	27 (50%)	25 (51.0%)	35 (68.6%)	21 (53.8%)
Lymph Nodes Total Number Mean ± Standard Deviation	404 7.9 ± 3.6	505 10.1 ± 5.4	315 6.4 ± 2.5	404 7.9 ± 2.6	134 3.7 ± 0.7

Abbreviations: CLL = chronic lymphocytic leukaemia; DLBCL = diffuse large B-cell lymphoma; FL = follicular lymphoma; MCL = mantle-cell lymphoma

Patients underwent baseline contrast-enhanced CT (CECT) or PET/CT at our institution between January 2012 and December 2020. For PET/CT, only examinations with co-registered diagnostic, contrast-enhanced, full-dose CT were included, while studies with low-dose, non-contrast attenuation-correction CT were excluded during screening.

Among 201 patients with NHL, 168 underwent diagnostic contrast-enhanced CT (CECT) alone and 33 underwent PET/CT with contrast-enhanced, full-dose CT (DLBCL *n* = 25, MCL *n* = 3, FL *n* = 2, CLL *n* = 3). PET/CT studies limited to low-dose, non-contrast attenuation-correction CT were excluded. Patients were followed for at least three years or until death, whichever occurred first. All analyses were conducted exclusively on CT imaging. Target lymph nodes were identified on diagnostic CT by pathological enlargement in accordance with the Cheson criteria [31].

For patients who underwent PET/CT, only the CT component was utilised; PET information was not required for node inclusion and was not used to confirm malignancy at the node level. In line with clinical practice, one biopsy-accessible index node per patient underwent histopathological verification and served as the reference standard, while all remaining segmented nodes were labelled by pathological size according to Cheson criteria.

**Fig. 1:**
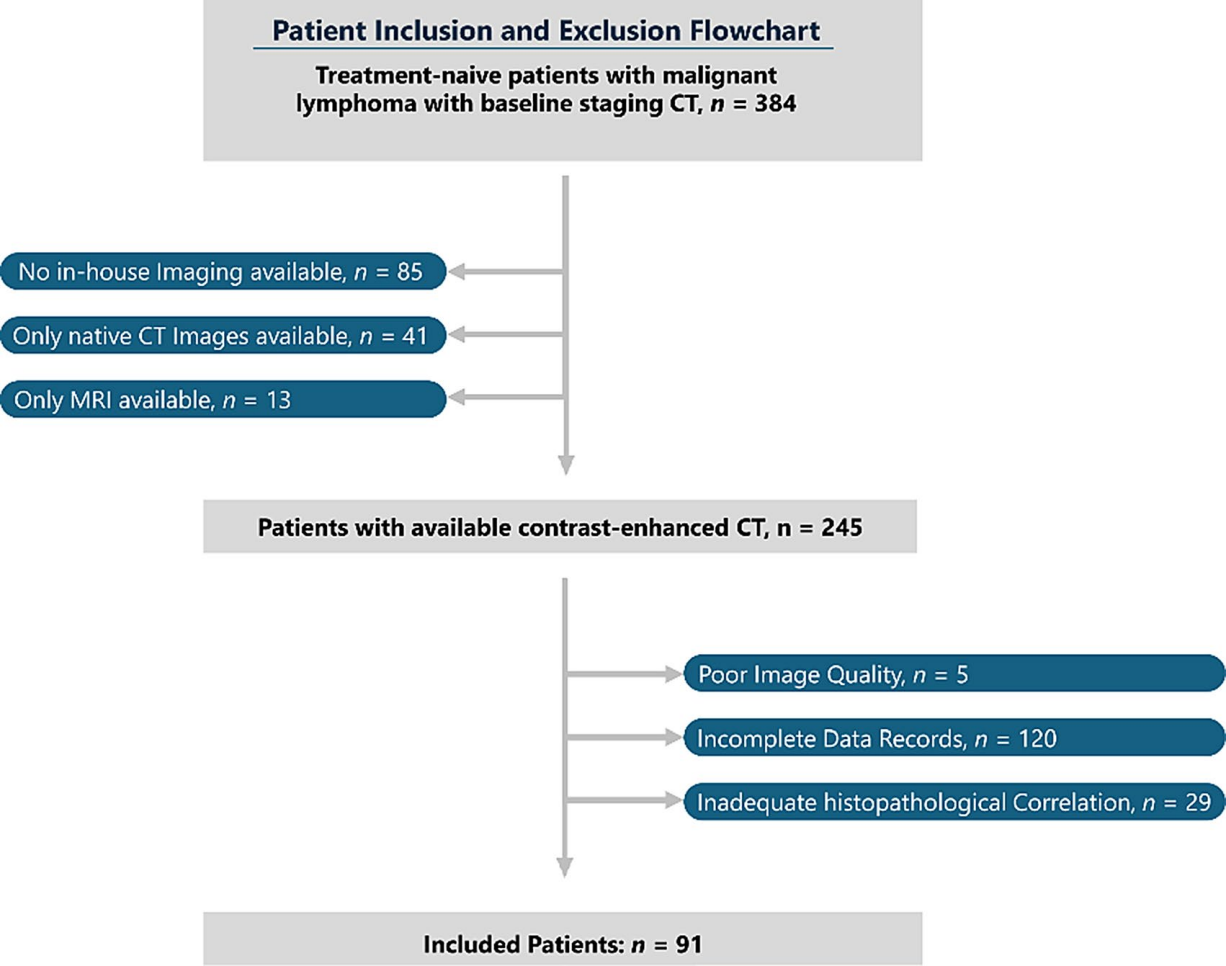
Recruitment pathway of the study

All scans subsequently underwent standardized preprocessing and feature extraction. Patients were excluded if they had incomplete clinical or imaging data after completion of first-line therapy, if histopathological confirmation was lacking, and if the first available in-house imaging was performed after the start of therapy. A flowchart of cohort selection is presented in Fig. 1.

In the lymphoma group, 1,628 target LNs were eligible for inclusion: the total number of LNs in CLL was *n = * 404 (mean number of LNs: 7.9 ± 2.6), in DLBCL *n = * 505 (mean 10.1 ± 5.4), in FL *n = * 315 (mean 6.4 ± 2.5), and in MCL *n = * 404 (mean 7.9 ± 3.6).

The reference group consisted of 39 treatment-naïve patients with non-small cell lung cancer (NSCLC; 18 women and 21 men; mean age 64.9 ± 8.5 years) with normal axillary LNs as confirmed by at least two PET/CECT scans.

Clinical data of at least 5 years were required to exclude the diagnosis of a hematological disease and/or a malignancy in the axillary region. The total number of LNs in the reference group was 134 (mean number of LNs: 3.4 ± 0.7).

### Imaging

Images were acquired during routine diagnostic procedures using a multi-row detector CT (Philips Brilliance CT 64/Philips Brilliance iCT 256, Philips Healthcare, Best, The Netherlands; Siemens Somatom Definition AS/Siemens Edge Plus, Siemens Healthineers, Erlangen Germany).

The standard baseline CT staging protocol included a contrast-enhanced neck, chest, and abdomen series. All CECT scans were acquired after intravenous contrast injection at a weight-matched dose (tube voltage 100 kV-120 kV with automatically calculated tube current, matrix 512 × 512, in-plane resolution between 0.62 × 0.62 mm and 0.86 × 0.86 mm, slice thickness 1.0–5.0 mm).

The CT images of the [^18^F]-Fluorodeoxyglucose (18F-FDG) PET/CT scans (Discovery-LS, GE Medical Systems, Chicago, IL, USA; Biograph mCT 40, Siemens Healthineers, Erlangen Germany) were performed from the base of the skull to the upper thigh using the following parameters: 100–120 kVp with automatically calculated tube current, scan width of 4.0 mm reconstructed slice thickness with overlap of 3.0 mm. The CT images were reconstructed using three-dimensional (3D) CT attenuation correction with standard 512 × 512 filtered back projection reconstruction with a field of view (FOV) of 50–70 cm. All radiomic features were extracted from the portal-venous phase of diagnostic CECT only.

Complete response (CR) was defined as being in clinical remission with no residual morphology on CECT or PET/CT scans. Imaging was performed close to the start of treatment (maximum of 3 weeks) to assess the disease status.

### Segmentation and radiomic feature extraction

Having chosen approximately 8 lymph nodes (LNs) per patient in the lymphoma group and 3–4 LNs in the reference group as target lesions, segmentation was semi-automatically performed by one radiologist (CL, 10 years of experience in oncological radiology) [31]. Supplemental Table S1 lists the settings of the radiomics feature extraction. The segmentation of the 3D region of interest (ROI), the texture analysis, and the feature extraction were performed using the Mint Lesion™ software (version 3.8.4; mint Medical GmbH, Heidelberg, Germany).

After loading the Digital Imaging and Communications in Medicine (DICOM) files (entire image stack of CT data in portal-venous contrast phase), each individual LN (*n =* 1,762 LNs in 240 patients) was entirely segmented on each slice showing the LN with a semi-automatic tool and subsequent manual fine adjustments, allowing for precise 3D LN segmentation. Finally, radiomic features were quantified by analyzing distinct grey level patterns within each ROI. Textural feature descriptors were used following the guidelines of the Image Biomarker Standardisation Initiative (IBSI) [32].

From each ROI, 78 image features were extracted: features related to 3D size and shape, attenuation features, first-order features describing the distribution of voxel intensity within the selected region, and second-order features analyzing spatial relationships between voxels related to the grey-scale co-occurrence matrix (GLCM). Supplemental Table S2 lists the 78 extracted features.

### Feature selection and machine learning model development

To handle the intrinsic high-dimensionality of radiomics data we implemented a two-stage machine-learning pipeline that clearly separates feature selection from model training, thereby improving interpretability and limiting over-fitting.

### Stage 1 – Random-Forest feature triage

**Fig. 2:**
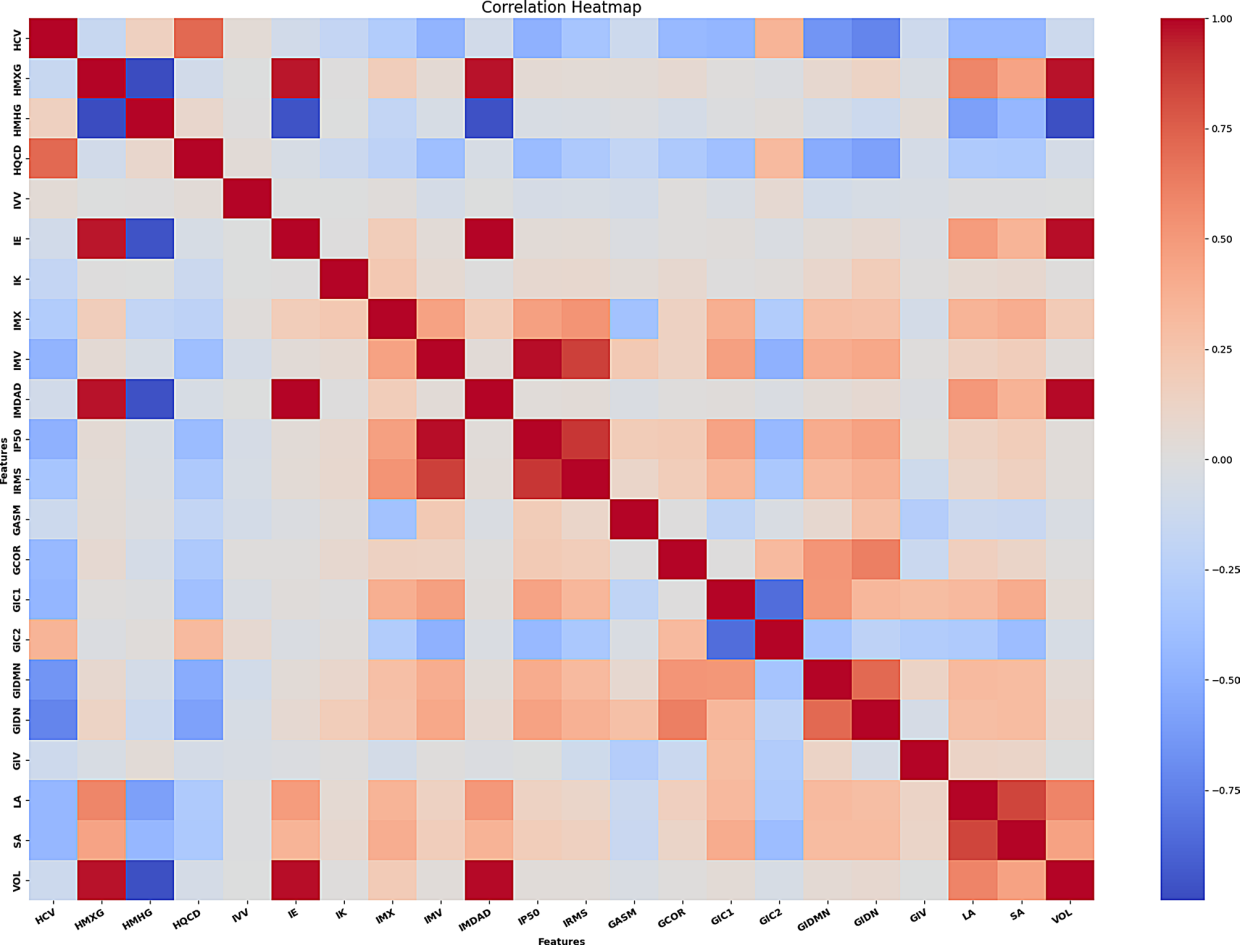
Heatmap of pair-wise Pearson correlations after consolidating percentiles to a single representative descriptor (IP50). Distinct clusters and isolated darker tiles mark occasional high correlations, whereas the overall light background indicates that most feature pairs show only weak association. This pattern confirms minimal redundancy within the selected feature set. A side-by-side comparison with the unconsolidated model is provided in Supplementary Fig. S2b

All extracted variables were first screened with a Random Forest (RF) algorithm, used exclusively for dimensionality reduction. RF ensemble trees rank predictors by their mean decrease in impurity, capturing non-linear interactions and remaining robust in the presence of noise and collinearity (Breiman 2001). Using scikit-learn’s SelectFromModel utility (Pedregosa et al. 2011; van Rossum & Drake 2009), we retained only those variables whose importance exceeded the data-driven threshold recommended by the library. This procedure, widely endorsed in radiomics (Parmar et al. 2015; Zwanenburg et al. 2020), reduced the initial feature set to 25 highly informative descriptors (see Supplemental Table S3). To ensure these 25 features were not merely relabeled versions of the same signal, we calculated pair-wise Pearson coefficients and displayed them in a heatmap (see Fig. 2). The heat-map thus serves as a qualitative cross-check on the RF filter: it confirms that most overt redundancy has been eliminated and that the surviving features form a largely orthogonal set. Together, Random Forest-based importance ranking and correlation analysis minimise multicollinearity and enhance the stability and interpretability of the downstream LGBM classifier.

### Stage 2 – Multiclass light gradient boosting machine

The reduced feature matrix was then fed into a single Light Gradient Boosting Machine configured for simultaneous five-class prediction (non-lymphoma, CLL, MCL, DLBCL, FL). LightGBM’s histogram-based, leaf-wise boosting delivers fast training and strong in-built regularisation, attributes well suited to structured radiomics data (Ke et al. 2017; Alzamzami et al. 2020). Running the algorithm in true multiclass mode learns all decision boundaries in one optimisation pass, preserving inter-class relationships and sidestepping the pitfalls of piecemeal pair-wise modelling.

### Software implementation and reproducibility

Both the RF-based SelectFromModel step and the LGBM classifier were coded in Python 3.10 using the scikit-learn and LGBM libraries, wrapped into custom scripts that automate preprocessing, ten-fold cross-validation and model persistence. The computational set-up—including library versions, Random-Forest parameters and the full set of LGBM hyper-parameters—is summarised in Supplemental Table S4.

### Statistical analysis

To maximise the generalisability of our results, we applied stratified 10-fold cross-validation throughout. The dataset was repeatedly partitioned into training (90%) and test (10%) subsets, with stratification performed at the patient level so that all lymph nodes from a given patient appeared in only one split. For each fold, we calculated the area under the ROC curve (AUC), accuracy, precision, recall and Specificity; final values are reported as the mean ± standard deviation across the ten test runs.

Receiver-operating-characteristic (ROC) curves were generated using a one-versus-the-rest strategy to evaluate performance both for the global lymphoma-versus-non-lymphoma task and for each subtype. To visualise class-specific errors, we also constructed a confusion matrix for the final LGBM model, indicating how often each subtype—and the non-lymphoma reference group—was correctly or incorrectly classified.

## Results

### Patient characteristics

In total, 201 consecutive patients with biopsy-confirmed malignant lymphoma—51 CLL, 50 DLBCL, 49 FL and 51 MCL—met the study’s inclusion criteria. In addition, 39 patients with PET/CT-verified normal axillary lymph nodes were analysed as a non-lymphoma reference group. Table 1 shows the baseline demographics of the malignant lymphoma cohort and the non-lymphoma cohort.

### Radiomic feature selection and machine learning modelling

From the contrast-enhanced CT images, 72 IBSI-compliant radiomic features were initially extracted. A Random-Forest (SelectFromModel) step reduced the initial feature set to 25 predictors for downstream modelling. Redundancy was assessed via pairwise Pearson correlations (heatmap, Fig. 2). To address the correlation among closely related intensity percentiles and improve parsimony, we replaced IP25/IP50/IP75/IP90 with the single representative descriptor IP50 prior to re-training. Correlation heatmaps before and after consolidation are provided in Supplementary Fig. S2a–b; predictive performance was essentially unchanged. Only isolated dark tiles indicate a few strong correlations; the predominantly pale background denotes low collinearity across the reduced feature matrix. This qualitative assessment corroborates the Random-Forest selection and supports a stable, interpretable model foundation. With redundancy minimized, we proceeded to model development. The RF-selected variables were entered into a single multiclass Light Gradient Boosting Machine (LGBM) classifier that simultaneously performed (i) lymphoma-versus-non-lymphoma discrimination and (ii) four-way subtype assignment (CLL, MCL, DLBCL, FL).

**Fig. 3:**
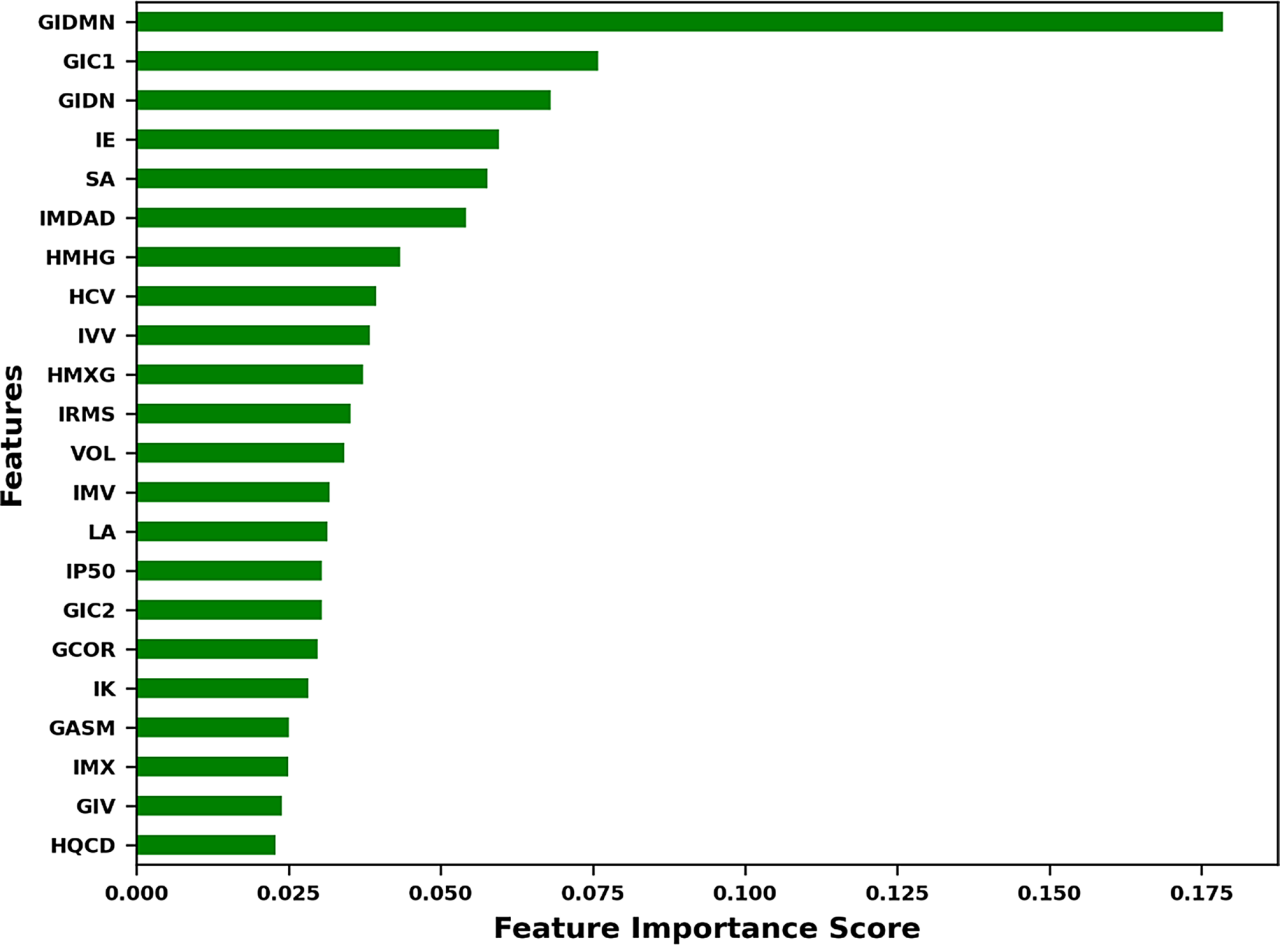
Feature-importance ranking after percentile consolidation (HP_median = IP50). Bars show the mean decrease in impurity for the final multiclass LGBM, indicating each predictor’s contribution to lymphoma–versus–non-lymphoma discrimination and subtype classification. A side-by-side comparison with the unconsolidated model is provided in Supplementary Fig. S3b. Detailed importance values for retained features are reported in Supplementary Table S3; the full catalogue of 78 assessed features is listed in Supplementary Table S2

**Fig. 4:**
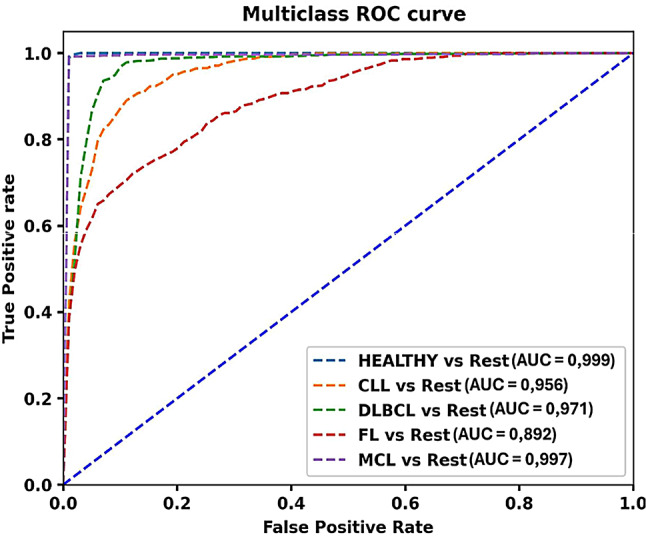
Multiclass ROC analysis after percentile consolidation demonstrates very high discrimination between lymphoma and non-lymphoma (AUC = 0.999). Among subtypes, MCL attains the highest AUC (0.997), followed by DLBCL (0.971) and CLL (0.956), whereas FL trails at 0.892. A side-by-side comparison with the unconsolidated model is provided in Supplementary Fig. S4b. Abbreviations: CLL = chronic lymphocytic leukaemia; DLBCL = diffuse large B-cell lymphoma; FL = follicular lymphoma; MCL = mantle-cell lymphoma

Predictor influence is illustrated in a feature-importance bar plot (see Fig. 3) where variables are ranked by mean decrease in impurity, giving an intuitive view of which descriptors shape each decision boundary. Discriminative performance was quantified using one-vs-rest multiclass ROC analysis for the overall lymphoma-versus-non-lymphoma task and for each subtype. Figure 4 shows the ROC curves for the consolidated model (IP50 only). A side-by-side comparison with the unconsolidated model (IP25/IP50/IP75/IP90) demonstrates near-perfect separation of non-lymphoma and lymphoma (see Supplementary Fig. S4a–b). Numerical results for the consolidated model are summarised in Table 2 (for the unconsolidated model in Supplementary Table S2b).

**Table 2:** Discriminative performance of the multiclass prediction model after percentile consolidation to classify lymphoma from non-lymphoma and lymphoma subtypes

Classification	AUC	Precision	Sensitivity/ Recall	F1-score	Specificity
Non-lymphoma vs. lymphoma	0.999 ± 0.001	0.890 ± 0.099	0.970 ± 0.041	0.924 ± 0.058	0.991 ± 0.005
MCL	0.997 ± 0.006	0.986 ± 0.023	0.980 ± 0.013	0.983 ± 0.013	0.997 ± 0.004
DLBCL	0.971 ± 0.016	0.911 ± 0.040	0.934 ± 0.053	0.921 ± 0.033	0.955 ± 0.024
CLL	0.956 ± 0.014	0.776 ± 0.113	0.827 ± 0.075	0.795 ± 0.063	0.941 ± 0.028
FL	0.892 ± 0.027	0.732 ± 0.106	0.637 ± 0.093	0.672 ± 0.061	0.955 ± 0.02

Abbreviations: AUC: Area under the curve, CLL = chronic lymphocytic leukaemia; DLBCL = diffuse large B-cell lymphoma; FL = follicular lymphoma; MCL = mantle-cell lymphoma

The consolidated model achieved an overall accuracy of 0.874 ± 0.021. In the one-vs-rest comparison, discrimination of non-lymphomatous (HEALTHY) nodes from lymphomatous nodes was near-perfect (AUC 0.999 ± 0.001; precision 0.890 ± 0.099; recall 0.970 ± 0.041; F1-score 0.924 ± 0.058; specificity 0.991 ± 0.005). Among subtypes, MCL performed highest (AUC 0.997 ± 0.006; precision 0.986 ± 0.023; recall 0.980 ± 0.013; F1 0.983 ± 0.013; specificity 0.997 ± 0.004), followed by DLBCL (AUC 0.971 ± 0.016; precision 0.911 ± 0.040; recall 0.934 ± 0.053; F1 0.921 ± 0.033; specificity 0.955 ± 0.024) and CLL (AUC 0.956 ± 0.014; precision 0.776 ± 0.113; recall 0.827 ± 0.075; F1 0.795 ± 0.063; specificity 0.941 ± 0.028). FL showed lower but acceptable performance (AUC 0.892 ± 0.027; precision 0.732 ± 0.106; recall 0.637 ± 0.093; F1 0.672 ± 0.061; specificity 0.955 ± 0.020).

**Fig. 5:**
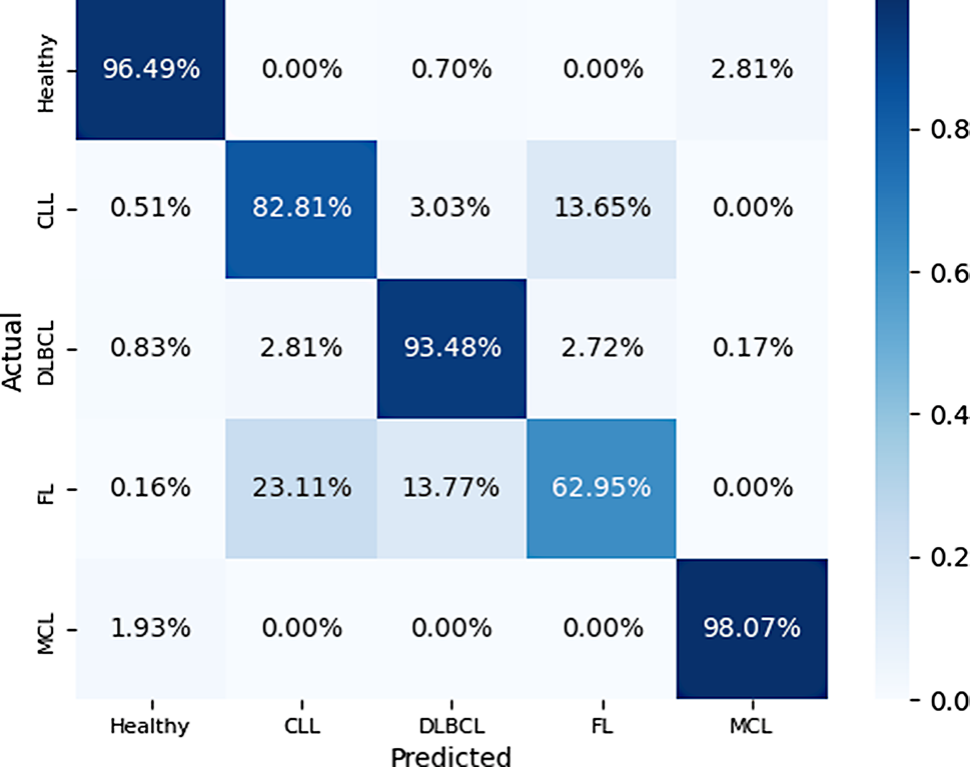
Confusion matrix for the multiclass LGBM model after percentile consolidation. Correct predictions dominate the diagonal for healthy nodes, MCL (98.1%), and DLBCL (93.5%). Most errors involve FL, which is mislabelled as CLL (23.1%) or DLBCL (13.8%); conversely, 13.7% of CLL cases are predicted as FL. A side-by-side comparison with the unconsolidated model is provided in Supplementary Fig. S5b. Abbreviations: CLL = chronic lymphocytic leukaemia; DLBCL = diffuse large B-cell lymphoma; FL = follicular lymphoma; MCL = mantle-cell lymphoma

The confusion matrix in Fig. 5 supports these findings. Correct predictions dominate the main diagonal for healthy nodes, MCL and DLBCL, whereas FL shows the greatest confusion: 23% of true FL cases are labelled as CLL and 14% as DLBCL.

Conversely, 14% of CLL cases are misclassified as FL. This pattern reflects the greater morphological and textural similarity of FL to indolent CLL and to less aggressive regions within DLBCL, and pinpoints FL as the principal source of residual ambiguity in the model.

## Discussion

The present study investigated whether radiomics-based machine-learning classifiers derived from routine CT and PET/CT can (i) separate lymphoma from non-lymphoma tissue and (ii) assign the correct histological subtype. Such tools could provide non-invasive imaging biomarkers early in the diagnostic pathway. Using a Random-Forest (RF) feature selection followed by a Light Gradient Boosting Machine (LGBM), we achieved excellent performance: the model distinguished lymphoma from non-lymphoma tissue and accurately classified the most prevalent non-Hodgkin lymphoma (NHL) subtypes, with the highest scores for mantle-cell lymphoma (MCL) and diffuse large B-cell lymphoma (DLBCL).

Rapid, precise subtyping is clinically critical because histology and baseline imaging together determine stage, treatment regimen and therapy timing [2]. Yet subtype assignment still relies on excising the most accessible lymph node for histopathology—an invasive procedure with associated morbidity [6].

Cross-sectional imaging, performed before, during and after therapy, already complements biopsy by mapping disease distribution and treatment response [33, 34]. Radiomics magnifies this value by extracting high-dimensional texture information invisible to the naked eye.

Radiomics-driven machine learning has proved versatile across modalities, differentiating malignant from benign lesions, lymphoma from non-lymphoma nodes, and individual lymphoma subtypes [26–28, 30, 35–37]. In lymphoma research, however, most studies focus on outcome prediction—especially in Hodgkin lymphoma and DLBCL—and rely largely on PET/CT [18, 38–41].

Few studies have tackled subtype classification, and normal lymph nodes have rarely served as negative controls [22–24, 42, 43]. Prior examples illustrate this gap. Lippi et al. analysed texture features from PET/CT in 60 patients—DLBCL (*n =* 9), FL (*n =* 9), Hodgkin’s lymphoma (*n =* 24) and MCL (*n =* 9)—and used a machine-learning pipeline to further classify these entities [42]. De Jesus et al. differentiated FL (*n =* 44) from DLBCL (*n =* 76) with radiomic PET/CT signatures [22], whereas Wu et al. demonstrated, on MRI, that textural statistics could separate 30 DLBCL from 11 FL cases [24]. Reinert et al. employed a CT-based radiomics model to distinguish 18 DLBCL Richter-transformation cases from 36 CLL patients, achieving an AUC of 0.85 [23]. Finally, Enke et al. extracted splenic radiomic features in 229 NHL patients—DLBCL (*n =* 129), FL (*n =* 52), MCL (*n =* 48) – together with 97 Hodgkin’s lymphoma and 56 non-lymphoma controls, and reported an overall subtype-classification AUC of 0.75 [43].

Other imaging modalities are likewise being explored for lymphoma characterisation; for example, Zhang et al. demonstrated that elastosonography augments conventional ultrasonography in distinguishing benign from malignant superficial lymphadenopathies [44].

In parallel, related multiparametric radiomics work—such as MRI-based prediction of Ki-67 expression in primary CNS lymphoma —reinforces the same trajectory [45]. Taken together, these advances point to a broader shift toward quantitative, modality-agnostic imaging biomarkers that refine lymphoma assessment while reducing diagnostic invasiveness, to which our CT-based radiomics approach contributes.

Within this context, our study contributes a large, balanced NHL cohort: 201 treatment-naïve patients—50 DLBCL, 51 MCL, 49 FL, 51 CLL—plus 39 controls with healthy nodes. The RF–LGBM framework outperformed previous reports, with AUCs of 0.997 for MCL, 0.971 for DLBCL and 0.956 for CLL, likely reflecting both training volume and the efficiency of the two-step pipeline [46–49].

The model’s pronounced ability to differentiate CLL from DLBCL suggests a translational opportunity. Prospective, longitudinal radiomics could characterise the textural evolution of indolent CLL toward aggressive phenotypes observed in Richter transformation, providing imaging correlates of biological progression and informing dynamic risk stratification.

The lower AUC for FL probably reflects biological subtlety: FL comprises small- to medium-sized neoplastic cells arranged in follicles that resemble normal germinal-centre lymphocytes, whereas DLBCL shows diffuse sheets of larger cells with more aggressive features and greater extracellular vascularity—differences that amplify texture contrasts relative to healthy lymph-node tissue [2, 3]. These intrinsic histopathological differences likely impact radiomic metrics and explain the performance disparity. Modern taxonomy further underscores intra-histotype heterogeneity. The WHO 5th edition lists nearly 100 mature B- and T-cell entities, many defined by recurrent genetic or epigenetic lesions [3].

Large-scale genomic studies show that each of the four histotypes investigated here—DLBCL, FL, CLL and MCL—splinters into multiple molecular subgroups with distinct biology, therapeutic vulnerabilities and outcomes [50–54].

Accordingly, our framework is conceived as a rapid triage tool rather than a replacement for biopsy-based molecular work-ups: image-derived signatures can flag nodes likely to harbour aggressive clones or transformation and guide targeted sampling, while final therapy decisions remain grounded in histopathology and molecular profiling [55, 56].

We recognise several limitations. First, the retrospective, single-centre design and modest sample size introduce selection bias. Second, the analysis was confined to imaging; immunohistochemical and genomic markers were not included, so recurrent genetic lesions with prognostic impact in DLBCL, FL, CLL and MCL could not be modelled. Third, clinically measured variables were excluded, though they might enhance performance. To provide each histotype with adequate learning signal and to avoid majority-class bias, we constructed a prevalence-balanced dataset (~ 50 cases per subtype), which does not mirror real-world epidemiology and may overestimate clinical performance. Another limitation concerns the definition of ground truth: per patient, one biopsy-accessible index node underwent histopathological verification and served as the reference standard; all remaining segmented nodes were labelled by pathological size according to Cheson criteria, and PET information—when available—was not used for node inclusion or labelling. Size-based surrogate labelling can introduce misclassification, so performance estimates should be interpreted with this caveat. External validation in broader, prevalence-skewed, multicentre cohorts is essential; prevalence-aware solutions (e.g., cost-sensitive loss, calibrated probabilities or thresholds matched to true incidence) should be tested to support robust deployment across diverse settings. Given the single-centre setting with uniform diagnostic CECT protocols, no cross-site harmonisation (e.g., ComBat) was required or applicable in this study. Overall, this is a proof-of-concept feasibility study. The reported metrics derive from internal, patient-level cross-validation; without an independent validation cohort, generalisability across populations, vendors and acquisition protocols remains uncertain. A prospective, multicentre validation is planned, with consecutive all-comers at real-world subtype prevalence and standardised acquisition across scanners/vendors, using these results to inform a pre-specified protocol assessing externally validated multiclass performance, calibration and clinical utility. Looking ahead, integrated multi-omics workflows that merge radiomics with molecular and clinical data should be prioritised. Translation will depend on seamless workflow integration and close radiology–haematology collaboration, ideally with automated radiomic scores generated in PACS, reviewed in real time by a multidisciplinary lymphoma board and embedded in harmonised follow-up schedules, so that imaging insights consistently inform patient care.

## Conclusion

Radiomics derived from routine imaging shows strong potential for non-invasively classifying DLBCL, CLL, FL and MCL. By accelerating subtype identification, guiding biopsy selection, and supporting longitudinal disease monitoring, these imaging biomarkers could aid physicians in decision making for advanced disease monitoring, with the potential for developing imaging biomarkers for thriving precision oncology.

## Supplementary Information

Below is the link to the electronic supplementary material.

Supplementary Material 1

## Data Availability

The data supporting this study are available from the corresponding author upon reasonable request.

## Data availability

The data supporting this study are available from the corresponding author upon reasonable request.

## Abbreviation

AUC: Area under the curve
CI: Confidence interval
CECT: Contrast-enhanced CT
CLL: Chronic lymphocytic leukemia
CT: Computed tomography
DICOM: Digital Imaging and Communications in Medicine
DLBCL: Diffuse large B-cell lymphoma
FL: Follicular lymphoma
FDG: Fluorodeoxyglucose
GLCM: Grey-level co-occurrence matrix
LN: Lymph node
MCL: Mantle cell lymphoma
MRI: Magnetic resonance imaging
NSCLC: Non-small cell lung cancer
NHL: Non-Hodgkin lymphoma
PACS: Picture archiving and communication system
PET: Positron emission tomography
ROC: Receiver operating characteristic
ROI: Region of interest
VOI: Volume of interest
WHO: World Health Organization
3D: Three-dimensional
