# Supplementary material for: Non-Hodgkin’s lymphoma classification using 3D radiomics machine learning models for precision imaging in oncology
Source: BMC Med Imaging. 2025 Oct 30;25:435. doi: 10.1186/s12880-025-02006-3 (PMC12577418; doi:10.1186/s12880-025-02006-3)
Supplement: Supplementary file 1 — Supplementary Material 1 [file 12880_2025_2006_MOESM1_ESM.docx]

## SUPPLEMENTAL MATERIALS

# **Supplemental Table S1**: Settings of the Radiomics Feature Extraction

**Settings of the Radiomics Feature Extraction**

Bin Method FBS

Bin Amount 20

LoG Filter 0

LoG Sigma 1

Matrix Aggregation Method 3D Average

Resample Filter 0

Resample Spacing X 1

Resample Spacing Y 1

Resample Spacing Z 1

Second-Order Distance 1

Threshold Filter 0

# **Supplemental Table S2**: Initial extracted radiomic features

**Initial extracted radiomic features**

**Basic Features**

Short axis

Long axis

Volume

Density-mean

Density-meanMinusSD

Density-meanPlusSD

**Radiomics Features of First Order:**

**Intensity-Based Features**

Intensity Variation

Intensity Energy

Intensity Interquartile Range

Intensity Kurtosis

Intensity Max

Intensity Mean

Intensity Mean absolute deviation

Intensity Median absolute deviation

Intensity Min

Intensity 10th percentile

Intensity 25th percentile

Intensity 50th percentile

Intensity 75th percentile

Intensity 90th percentile

Intensity Quartile coefficient dispersion

Intensity Range

Intensity Robust mean absolute deviation

Intensity Root mean square

Intensity Skewness

Intensity SD

Intensity Variance

**Intensity Histogram Features**

Histogram Coefficient variation

Histogram Entropy

Histogram Interquartile range

Histogram Kurtosis

Histogram Max

Histogram Maximum histogram gradient

Histogram Maximum histogram gradient intensity

Histogram Mean

Histogram Mean abs deviation

Histogram Median abs deviation

Histogram Min

Histogram Minimum histogram gradient

Histogram Minimum histogram gradient intensity

Histogram 10th percentile

Histogram 25th percentile

Histogram 50th percentile

Histogram 75th percentile

Histogram 90th percentile

Histogram Quartile coefficient dispersion

Histogram Range

Histogram Robust mean abs deviation

Histogram Skewness

Histogram SD

Histogram Uniformity

Histogram Variance

**Radiomics Features of Second Order:**

**Gray Level Co-Occurrence Matrix (GLCM) Features**

GLCM Angular second moment

GLCM Auto correlation

GLCM Cluster prominence

GLCM Cluster shade

GLCM Cluster tendency

GLCM Contrast

GLCM Correlation

GLCM Difference average

GLCM Difference entropy

GLCM Difference variance

GLCM Dissimilarity

GLCM Information correlation 1

GLCM Information correlation 2

GLCM Inverse difference

GLCM Inverse difference moment

GLCM Inverse difference moment normalized

GLCM Inverse difference normalized

GLCM Inverse variance

GLCM Joint average

GLCM Joint entropy

GLCM Joint maximum

GLCM Joint variance

GLCM Standard deviation

GLCM Sum of averages

GLCM Sum of entropy

GLCM Sum of variance

Radiomic feature extraction was performed by systematically analyzing grey-level intensity patterns within the defined volumes of interest (VOIs). To ensure methodological consistency and reproducibility, all texture features were computed in accordance with the standardized definitions and computational frameworks outlined by the Image Biomarker Standardisation Initiative (IBSI) [1].

# **Supplemental Table S3**: Random Forest-based selected Features

**Random Forest-based selected features**

| **Feature** | **Feature abbreviation** | **Importance score** |
| --- | --- | --- |
| GLCM Inverse difference moment normalized | GIDMN | 0.1614 |
| GLCM Information correlation 1 | GIC1 | 0.0827 |
| GLCM Inverse difference normalized | GIDN | 0.0571 |
| Short Axis | SA | 0.0528 |
| Intensity Energy | IE | 0.0510 |
| Intensity Median absolute deviation | IMDAD | 0.0504 |
| Histogram Maximum histogram gradient | HMXG | 0.0432 |
| Histogram Minimum histogram gradient | HMHG | 0.0383 |
| Histogram Coefficient variation | HCV | 0.0362 |
| Volume | VOL | 0.0338 |
| Intensity Variation | IVV | 0.0317 |
| GLCM Information correlation 2 | GIC2 | 0.0308 |
| Long Axis | LA | 0.0296 |
| Intensity Root mean square | IRMS | 0.0286 |
| GLCM Correlation | GCOR | 0.0272 |
| Intensity Mean | IMV | 0.0268 |
| Intensity 25th percentile | IP25 | 0.0266 |
| Intensity Kurtosis | IK | 0.0265 |
| GLCM Angular second moment | GASM | 0.0262 |
| Intensity 75th percentile | IP75 | 0.0255 |
| Intensity 90th percentile | IP90 | 0.0254 |
| GLCM Inverse variance | GIV | 0.0242 |
| Intensity Max | IMX | 0.0224 |
| Histogram Quartile coefficient dispersion | HQCD | 0.0213 |
| Intensity 50th percentile | IP50 | 0.0202 |

# **Supplemental Table S4: Model Development Details**

| Aspect | Details |
| --- | --- |
| Random Forest-based Feature Selection Parameters | 100 estimators, Gini impurity as split criterion, max_features='sqrt', random_state=0, default values for other hyperparameters. Parameters were found using GridSearchCV (5 Fold) (https://scikit-learn.org/stable/modules/generated/sklearn.model_selection.GridSearchCV.html). Feature selection was performed using SelectFromModel (https://scikit-learn.org/stable/modules/generated/sklearn.feature_selection.SelectFromModel.html).  ‘oob_score’ of RF was set to ‘False’. |
| Multiclass Machine Learning Model based on Light Gradient Boosting Machine (LGBM) | Hyperparameters: n_estimators: 100, learning_rate: 0.1,boosting_type: gbdt; objective: multiclass, min_child_samples: 20.  GridSearchCV was used  5-fold CV used for gridsearch to find best parameters |
| Validation Method | 10 Fold cross validation was performed for LGBM |
| Model Optimization | Grid search procedure used to fine-tune hyperparameters [2]. |
| Software & Implementation | Feature selection and model construction were implemented using the open-source Scikit-learn open-source Python machine learning library with the SelectFromModel" setting [3, 4]. PyCharm 2024.1.1, Scikit-learn version 1.3.0, http://scikit-learn.org/) |
| Computing Environment | Computing was performed on a single workstation running Ubuntu 20.04 LTS, equipped with 32GB RAM, an AMD Ryzen 9 3900X processor, and a Nvidia RTX 3080 10GB. |

**Model Development and Computational Setup**

# **Supplementary Fig. S2. Feature-correlation heatmaps before and after IP50**


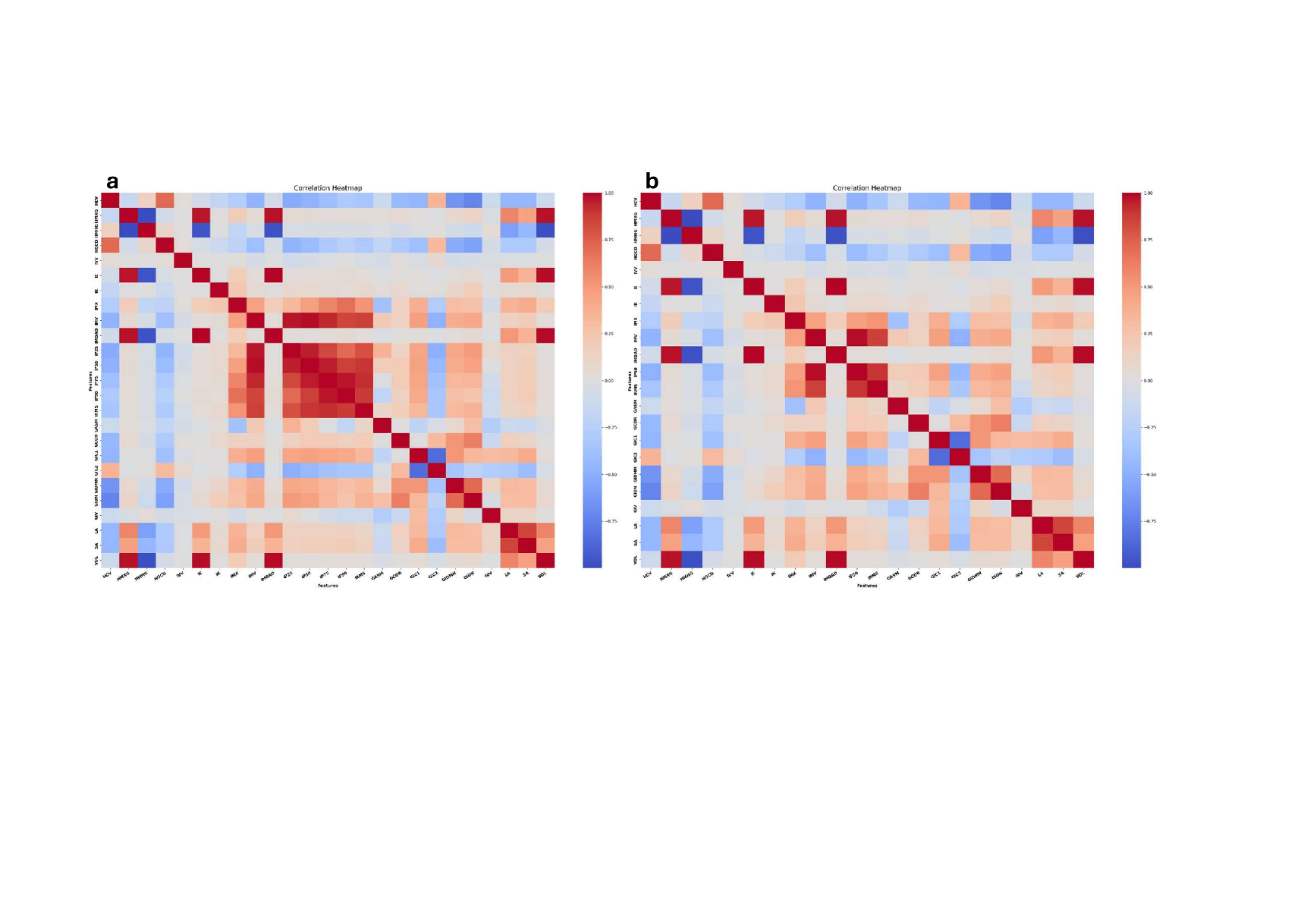
**consolidation**

**Figure 2.** Correlation structure before and after percentile consolidation.

(a) Pairwise Pearson correlations among the retained features when intensity percentiles IP25, IP50, IP75, and IP90 are included. Occasional darker tiles indicate isolated higher correlations, whereas the predominantly light background denotes generally weak associations.

(b) Correlation matrix after consolidating percentiles to a single representative descriptor (IP50). The overall pattern is essentially unchanged, indicating no noteworthy change in collinearity and supporting the parsimony of the IP50-consolidated feature set.

# **
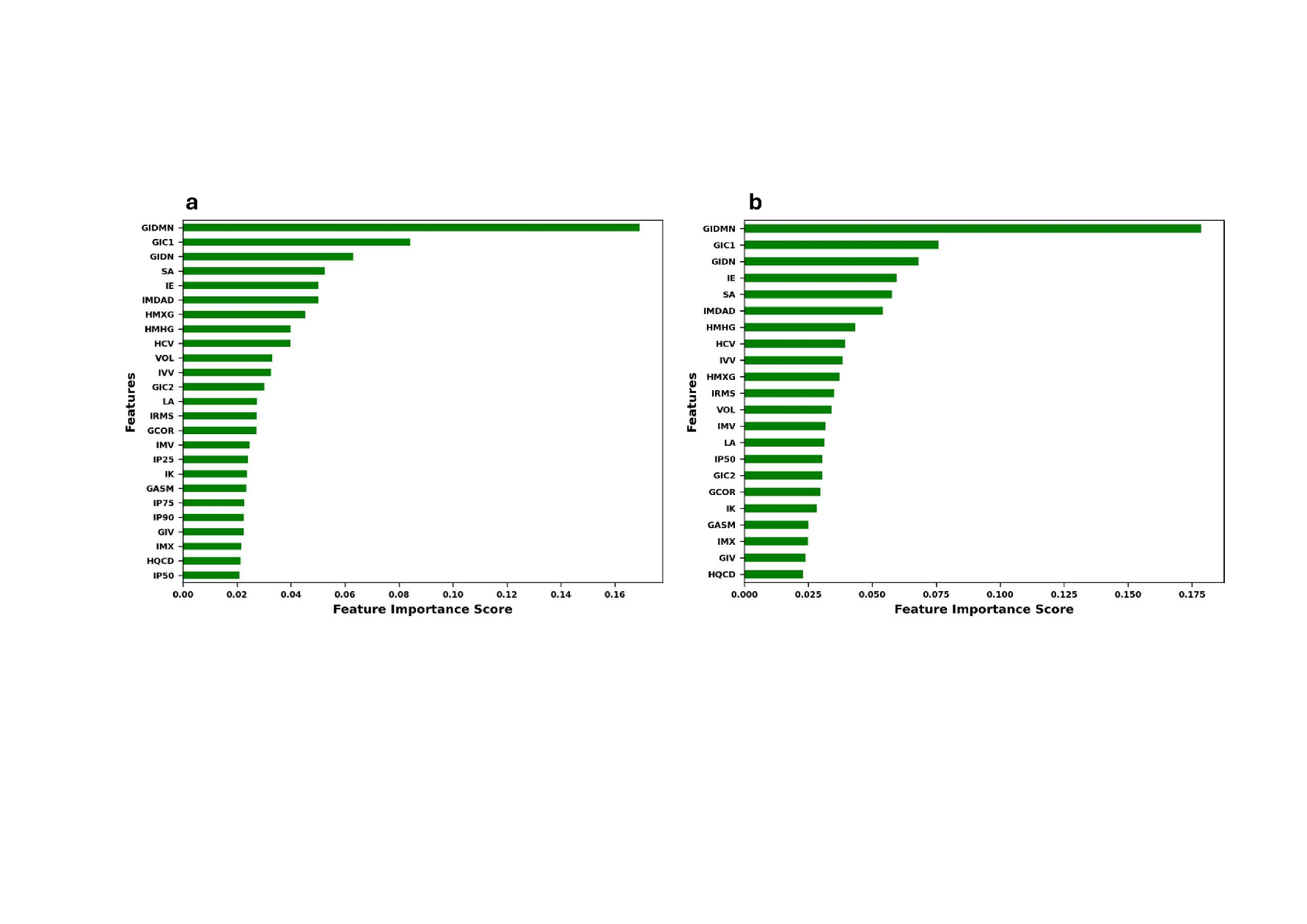
Supplementary Fig. S3. Feature-importance ranking before and after IP50 consolidation**

**Figure 3.** Feature-importance ranking for the radiomic predictors used in the LGBM model. Bars represent the mean decrease in impurity, illustrating each variable’s contribution to both lymphoma-versus-non-lymphoma separation and subtype discrimination. Across both panels, texture-derived descriptors dominate: GIDMN, GIC1, and GIDN remain the top contributors, followed by first-order/shape terms (e.g., IE, SA, IMDAD, HCV, VOL, LA). Percentile features sit in the lower–mid range.

In the unconsolidated model (a), IP25 occupies a mid-ranked position (with IP75/IP90 nearby). After percentile consolidation (b), IP50 appears at essentially the same relative rank as IP25 previously, indicating that pooling the percentiles into a single descriptor preserves the percentile signal without materially altering the hierarchy of predictors.

#
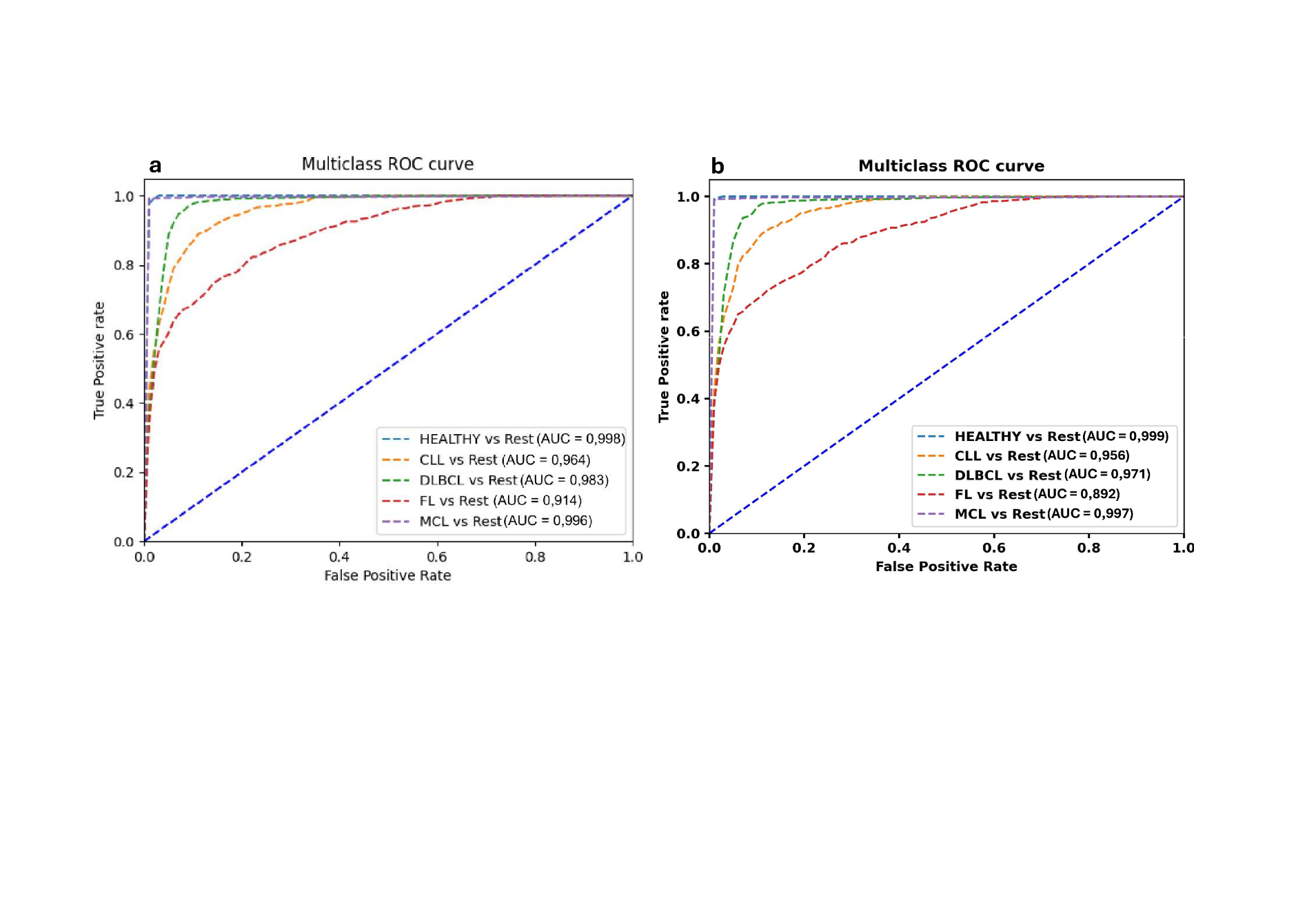
**Supplementary Fig. S4. Multiclass ROC curves before and after IP50 consolidation**

# **Figure 4.** Multiclass ROC curves before and after percentile consolidation.

# (a) Unconsolidated model (IP25/IP50/IP75/IP90).

# (b) Consolidated model (IP50 only). Discrimination of non-lymphoma vs. lymphoma remains near-perfect in both panels (AUC ≈ 0.999 vs 0.998). Subtype AUCs are essentially unchanged after consolidation: MCL highest (AUC ≈ 0.997 vs 0.996), followed by DLBCL (AUC ≈ 0.971 vs 0.983) and CLL (AUC ≈ 0.956 vs 0.964), with FL lower (AUC ≈ 0.892 vs 0.914). Curves are one-vs-rest.

# Abbreviations: CLL, chronic lymphocytic leukaemia; DLBCL, diffuse large B-cell lymphoma; FL, follicular lymphoma; MCL, mantle-cell lymphoma.

#
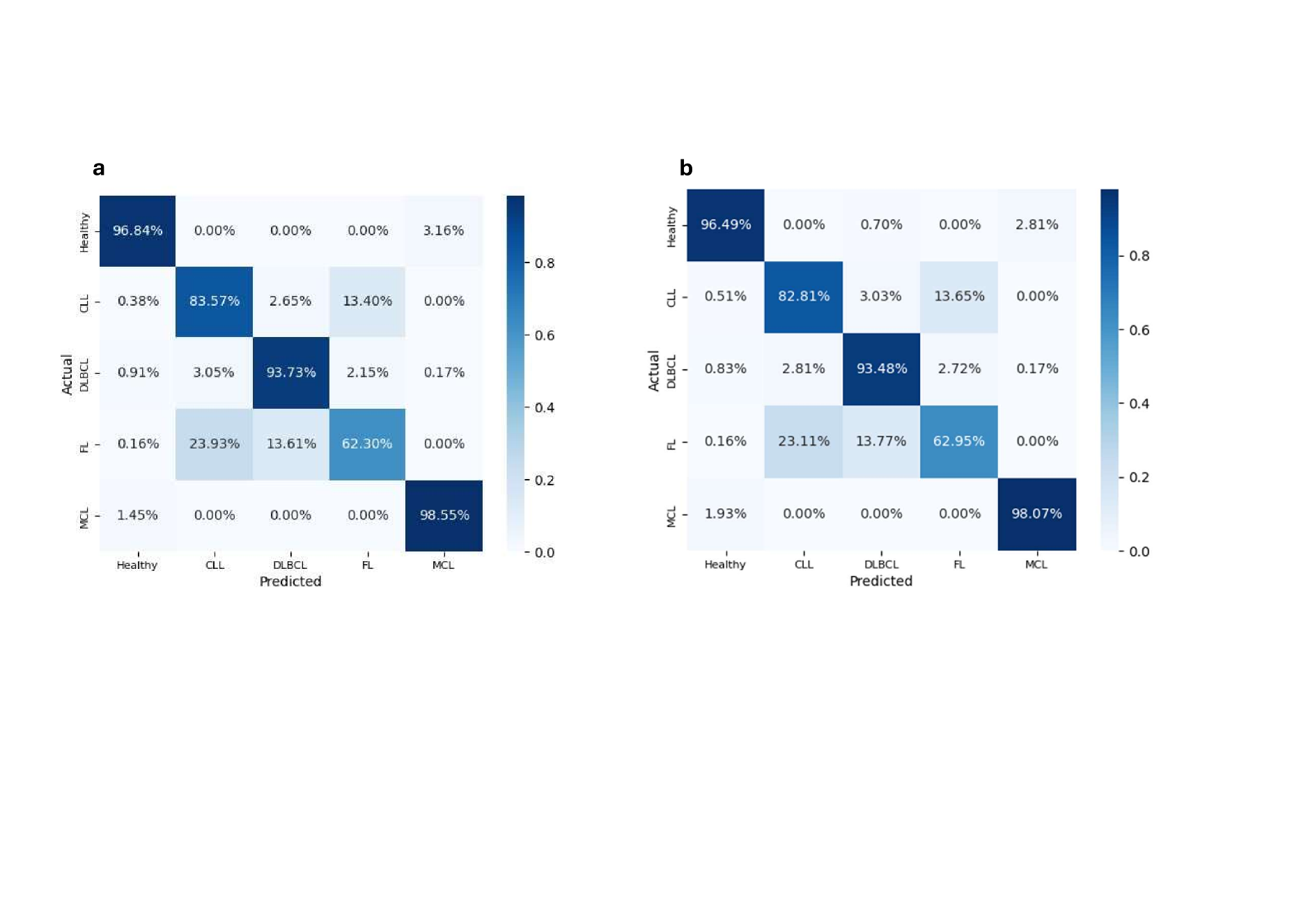
**Supplementary Fig. S5. Confusion matrix before and after IP50 consolidation**

# **Figure 5.** Confusion matrix before and after percentile consolidation. (a) Unconsolidated model (IP25/IP50/IP75/IP90). Correct predictions dominate the diagonal: Healthy 96.8%, MCL 98.6%, DLBCL 93.7%, CLL 83.6%, FL 62.3%. Most errors involve FL misclassified as CLL 23.9% or DLBCL 13.6%; CLL → FL 13.4%.

# (b) Consolidated model (IP50 only). The pattern is essentially unchanged: Healthy 96.5%, MCL 98.1%, DLBCL 93.5%, CLL 82.8%, FL 63%. FL remains the principal source of confusion (FL → CLL 23.1%, FL → DLBCL 13.8%), with a similar reciprocal error (CLL → FL 13.7%).

# Abbreviations: CLL, chronic lymphocytic leukaemia; DLBCL, diffuse large B-cell lymphoma; FL, follicular lymphoma; MCL, mantle-cell lymphoma.

# **Supplementary Table 2b:** Discriminative performance of the multiclass prediction model before percentile consolidation to classify lymphoma from non-lymphoma and lymphoma subtypes.

| **Classification** | **AUC** | **Precision** | **Sensitivity/ Recall** | **F1-score** | **Specificity** |
| --- | --- | --- | --- | --- | --- |
| Non-lymphoma vs. lymphoma | 0.998 ± 0.003 | 0.887 ± 0.098 | 0.970 ± 0.041 | 0.924 ± 0.058 | 0.993 ± 0.003 |
| MCL | 0.996 ± 0.005 | 0.986 ± 0.023 | 0.981 ± 0.012 | 0.983 ± 0.013 | 0.996 ± 0.003 |
| DLBCL | 0.983 ± 0.016 | 0.918 ± 0.036 | 0.937 ± 0.049 | 0.929 ± 0.030 | 0.957 ± 0.02 |
| CLL | 0.964 ± 0.016 | 0.771 ± 0.105 | 0.830 ± 0.080 | 0.798 ± 0.054 | 0.936 ± 0.027 |
| FL | 0.914 ± 0.027 | 0.729 ± 0.114 | 0.632 ± 0.086 | 0.680 ± 0.067 | 0.957 ± 0.019 |

**References:**

1. Zwanenburg A, Vallières M, Abdalah MA, Aerts HJ, Andrearczyk V, Apte A, et al. The image biomarker standardization initiative: standardized quantitative radiomics for high-throughput image-based phenotyping. Radiology. 2020;295:328–38.

2. Agrawal T. Hyperparameter Optimization Using Scikit-Learn. In: Hyperparameter Optimization in Machine Learning: Make Your Machine Learning and Deep Learning Models More Efficient. Berkeley, CA: Apress; 2021. p. 31–51.

3. Pedregosa F, Varoquaux G, Gramfort A, Michel V, Thirion B, Grisel O, et al. Scikit-learn: Machine learning in Python. the Journal of machine Learning research. 2011;12:2825–30.

4. van Rossum G, Drake FL. Python/C API Manual-Python 2.6. 2009.
